# Calcium Hydroxylapatite for Buttock Augmentation: A Global Expert Consensus on Best Practices and Treatment Considerations

**DOI:** 10.1111/jocd.71123

**Published:** 2026-08-19

**Authors:** Frank Wojan, Z. Paul Lorenc, K. Kay Durairaj, Gabriela Casabona, Julieta Spada, Tessa Cervantes, Stefania Guida, Samer Al Jalbout, Ross Radusky

**Affiliations:** ^1^ Merz Aesthetics Raleigh North Carolina USA; ^2^ Lorenc Aesthetic Plastic Surgery Center New York New York USA; ^3^ A Medical Corporation Pasadena California USA; ^4^ Ocean Clinic Marbella Spain; ^5^ Spada Dermatology and Aesthetics Buenos Aires Argentina; ^6^ LIV Dermatology and Aesthetics San Antonio Texas USA; ^7^ School of Medicine Vita‐Salute San Raffaele University Milan Italy; ^8^ Dermatology Clinic IRCCS San Raffaele Scientific Institute Milan Italy; ^9^ Private Practice Ravenna Italy

**Keywords:** biostimulatory, buttock augmentation, CaHA, calcium hydroxylapatite‐carboxymethylcellulose, contour, non‐surgical BBL, Radiesse

## Abstract

**Background:**

Minimally invasive body‐contouring procedures continue to increase in popularity as patients seek natural‐appearing enhancement with reduced downtime. Calcium hydroxylapatite‐carboxymethylcellulose (CaHA‐CMC; Radiesse) is a biostimulatory filler with established safety and effectiveness across approved facial, décolleté, and hand indications, and emerging evidence suggests potential benefit for improving buttock contour.

**Aims:**

To develop a global expert consensus outlining best practices for the use of CaHA‐CMC in buttock augmentation, including guidance on patient selection, aesthetic assessment, injection technique, safety considerations, and treatment planning.

**Methods:**

An international, multidisciplinary panel of aesthetic experts convened to develop consensus recommendations for CaHA‐CMC in buttock augmentation. Published clinical evidence was summarized, supplemented with real‐world expert experience, and recommendations were refined through interactive panel discussion.

**Results:**

Experts agreed that available evidence and clinical experience support the safe and effective use of undiluted and diluted CaHA‐CMC for buttock augmentation when administered by trained practitioners. Key recommendations include prioritizing contour correction over volumization; using a 1:1 CaHA‐CMC: saline dilution for optimal distribution and biostimulation; administering treatment across three sessions; and employing cannula‐based techniques with anatomically mapped injection zones. Across available studies and clinical experience, CaHA‐CMC produced consistent aesthetic improvements with a low incidence of adverse events.

**Conclusion:**

This global consensus provides practical, evidence‐informed guidance for CaHA‐CMC‐based buttock augmentation. Available evidence suggests that when applied with appropriate practitioner training and patient‐centered planning, CaHA‐CMC has a favorable safety profile and yields natural‐looking, reproducible improvements and predictable results in buttock contour. Standardized protocols and continuing outcomes research will further refine clinical practice.

## 1 Introduction

Evolving aesthetic preferences have shifted demand toward minimally invasive methods for buttock augmentation [1]. Nonsurgical interventions, including dermal fillers, offer shorter recovery times and lower risk when compared to surgical procedures that result in higher risk, adverse events, and mortality [1]. One nonsurgical option is calcium hydroxylapatite (CaHA‐CMC; Radiesse; Merz North America Inc., Raleigh, NC), a commercially available biostimulatory dermal filler composed of CaHA microspheres suspended in carboxymethylcellulose gel and glycerol [2]. As a regenerative dermal filler, CaHA‐CMC provides immediate volumetric correction in its undiluted form, while diluted formulations are used primarily to promote longer‐term biostimulatory effects rather than direct volumization [3, 4, 5]. Radiesse is well established as safe and effective within its approved indications, including correction of facial wrinkles and folds, improvement of décolleté wrinkles, improvement in loss of jawline contour, and correction of volume loss in the back of the hands [2, 6, 7, 8, 9].

Demand for natural, minimally invasive options has prompted broader CaHA‐CMC use, with buttock enhancement as a frequently requested procedure [10]. Interest in body contouring has likely been influenced by multiple factors, including increasing adoption of weight‐loss therapies, greater recognition of post‐weight‐loss soft‐tissue changes, and the influence of social media and wellness trends [11, 12]. Recent surveys of aesthetic healthcare professionals indicate that patients receiving GLP‐1 agonists commonly seek treatment for skin laxity and volume loss following weight reduction [13]. However, the extent to which GLP‐1‐associated weight loss has specifically increased demand for buttock augmentation has not been established. These observations highlight the need for nonsurgical treatments targeting aesthetic parameters, including contour, skin tightening, and skin quality, that can be concurrently addressed via biostimulation and not traditional volumizing fillers.

The combined mechanisms of CaHA‐CMC—immediate volume replacement coupled with longer‐term dermal remodeling—render it a plausible modality for improving buttock contour, shape, and skin quality [3, 4]. However, published and peer‐reviewed clinical evidence supporting CaHA‐CMC for buttock enhancement remains limited, with reports predominantly comprising small observational studies, case reports, and case series (Table 1). These findings support the potential utility of CaHA‐CMC for buttock enhancement but also emphasize the need for more robust clinical evaluation and further clinical studies.

**TABLE 1:** Published clinical data of CaHA‐CMC in buttock augmentation.

Citation	Condition targeted	Patients	Study design	Regimen for buttock augmentation	Result summary
Bartsch et al. [14]	Skin laxity and cellulite in buttocks and thighs	61 females	Prospective, randomized, clinical study	3.0 mL CaHA‐CMC (1:1); 6 mL total; 3 mL/side CaHA‐CMC administered in 3 treatment arms: TSGS (Day 0) → CaHA‐CMC (Day 90) TSGS (Day 0) → MFU‐V + CaHA‐CMC (Day 90) MFU‐V + CaHA‐CMC (Day 0) → TSGS (Day 90)	All arms demonstrated significant (*p* < 0.001) improvement in skin laxity and dimpling at Month 9, as assessed by patients, treating investigators, and independent, blinded evaluators. Procedural pain was noted for TSGS and MFU‐V; no AEs requiring medical intervention were observed.
Casabona [15]	Skin laxity and cellulite in buttocks and thighs	20 females	Retrospective, evaluator‐blind study	1.5 mL CaHA‐CMC (1:1); 6 mL total; 3 mL/side following MFU‐V at Day 0	Significant (*p* < 0.001) improvement was noted in cellulite appearance and skin laxity, as assessed by blinded evaluators using the CSS, at Day 90. Patients reported they were very satisfied (*n* = 10) or satisfied (9) with overall outcomes (One patient reported no change in dimpling). Treatment was well tolerated, with only mild, transient AEs reported, including pain (*n* = 20), bruising (18), edema (10), injection‐site induration (5), and erythema (2).
Casabona [16]	Skin laxity in buttocks	2 females	Case report	Patient 1: 13.5 mL CaHA‐CMC (1:1); 27 mL total; 13.5 mL/side across 3 treatment sessions at Day 0, 90, and 180, and following MFU‐V at Day 0 Patient 2: 15 mL CaHA‐CMC (1:1 & 1:2); 42 mL total; 21 mL/side across 2 treatment sessions at Day 0 and 90 and following MFU‐V at Day 0	At 3 months after last treatment, a 2‐grade improvement was observed in both patients according to the Gluteus Laxity Score [17] (It is assumed, because not stated otherwise, that the treating investigator performed the scale ratings).
Durairaj et al. [18]	Cellulite in buttocks	24 females	Prospective, open‐label, investigator‐initiated study	18 mL CaHA‐CMC (1:1); 36 mL total; 18 mL/side across 3 sessions at Week 0, 4, and 8	Significant (*p* < 0.0001) improvement was reported in CSS scores and number of visible dimples. By qualitative photo assessment, dimple depth was improved compared to baseline. Investigators and patients reported substantial improvement in cellulite appearance using the Global Aesthetic Improvement Scale at Week 14. Patients reported satisfaction with their aesthetic appearance and with their overall treatment. Minimal to no discomfort was reported, and no serious complications were observed. Transient injection‐site AEs included bruising (8.3%) and hemosiderin staining (4.1%).
Niaz et al. [19]	Cellulite in buttocks	1 female	Case report	6 mL CaHA‐CMC (1:2); 18 mL total; 9 mL/side in 1 treatment session	At Week 8, patient reported significant improvement in cellulite appearance and buttock dimples with a mild lift. Treatment was well tolerated with mild ecchymosis at the point of entry.
Silveira & Martinez [20]	Contour and volume in buttocks	3 females	Case series	30 mL CaHA‐CMC (1:6); 200 mL total; 100 mL/side in 1 treatment session	Resulted in a volumized, non‐cellulitic, symmetrical, and natural‐looking gluteal augmentation, with patient‐desired outcomes in hip dips, projection, and contouring.
Teodoro [21]	Contour, volume, skin laxity, and cellulite in buttocks	6 females, 1 male	Case series	Up to 30 mL CaHA‐CMC (1:1, 1:2, & 1:4); 117 mL total and dictated by patient case in 1 treatment session CaHA‐CMC ranges per condition: Cellulite: 1.5 to 6 mL (1:1) Contour: 4.5 to 12 mL (1:2) Volume: 18 mL (1:2) Laxity: 4.5 to 15 mL (1:4)	Patients reported being highly satisfied as early as Day 90. Minimal procedural‐related pain and discomfort were reported, and no AEs occurred.

Abbreviations: AEs, adverse events; CSS, Cellulite severity scale [22]; MFU‐V, Microfocused ultrasound with visualization; TSGS, Tissue‐stabilized guided subcision.

This global expert consensus integrates available clinical evidence with perspectives from real‐world practice to provide evidence‐informed guidance for the use of CaHA‐CMC in buttock augmentation. Because published evidence remains limited and is derived primarily from a small number of prospective and retrospective studies, case reports and case series, and expert experience, several recommendations reflect expert consensus rather than high‐level comparative evidence.

## 2 Methods

An international group of seven clinical experts convened virtually in October 2025 to develop consensus‐based recommendations regarding the safe and effective use of CaHA‐CMC in buttock augmentation. Panel members were purposively selected based on their recognized expertise in aesthetic medicine, extensive clinical experience with CaHA‐CMC and buttock contouring procedures, academic contributions to the field, and geographic representation across North America, Europe, and South America. The panel included dermatologists and plastic surgeons from Argentina, Italy, Spain, Brazil, and the United States, representing both private and academic practice settings. Panel characteristics and experience are provided in Table 2.

**TABLE 2:** Professional characteristics of consensus panel members.

Characteristic	Panel summary
Panel members, *n*	7
Degrees, *n* (%)	
MD	6 (85.7)
MD/PhD	1 (14.3)
Primary specialty, *n* (%)	
Dermatology	5 (71.4)
Plastic surgery	2 (28.6)
Years in aesthetic practice	Mean 19 (range, 11–30)
Years of experience with Radiesse^a^	Mean 14 (range, 9–20)
Years of experience with buttock treatment	Mean 14 (range, 4–30)
Practice setting, *n* (%)	
Private practice	5 (71.4)
Academic or mixed practice	2 (28.6)
Geographic distribution	North America, Europe, South America

^a^ Years of experience with Radiesse reflect self‐reported clinical use of calcium hydroxylapatite‐based injectables and are presented to provide context for panel expertise relevant to the consensus topic.

Relevant clinical literature was identified via PubMed search (keywords: calcium hydroxylapatite, buttock augmentation, and gluteal enhancement), with additional sources identified through reference‐list review (Table 1).

This project used a structured expert consensus process and did not employ a formal consensus methodology such as the Delphi, modified Delphi, or RAND/UCLA Appropriateness Method. The process consisted of one structured virtual meeting followed by up to three rounds of electronic manuscript review and revision. Before the meeting, panelists reviewed the relevant published literature and key discussion topics. During the meeting, consensus recommendations were developed through structured expert deliberation, with published evidence reviewed alongside the panelists' individual and collective clinical experience. There was no formal voting. Inclusion of consensus statements in the final manuscript required unanimous agreement among all seven panel members. Recommendations that did not initially achieve unanimous agreement were revised and further refined during the manuscript review process until consensus was achieved.

Given the limited availability of randomized controlled trials and comparative studies in buttock contouring, many recommendations represent expert consensus informed by the published literature and extensive clinical experience. The resulting consensus recommendations address optimal treatment techniques, patient selection, and practical considerations for safe and effective CaHA‐CMC‐based buttock augmentation.

## 3 Results

Unless otherwise indicated, the recommendations presented below represent expert consensus informed by the available published evidence and the panel's clinical experience. Where supporting evidence is limited, recommendations should be interpreted as expert guidance rather than definitive evidence‐based practice.

Consensus statement recommendations proposed by the expert panel are summarized in Table 3. These recommendations are based primarily on expert consensus informed by a small number of prospective and retrospective studies and published clinical experience unless otherwise indicated.

**TABLE 3:** Panel consensus statements for CaHA‐CMC in buttock augmentation.^a^

**Overall consensus**	
When performed with proper training and education, both undiluted and diluted CaHA‐CMC (Radiesse) are safe and effective for injection into the buttocks to achieve buttock augmentation.	
**General statements**	
CaHA‐CMC	As a regenerative dermal filler, CaHA‐CMC offers both immediate and long‐lasting biostimulatory volumization.
	CaHA‐CMC is safe and effective when used as a dermal filler for approved indications (face, hand, décolleté and jawline), and increasing evidence supports its potential utility in gluteal augmentation.
Buttock treatment	Based on available evidence and expert consensus, the panel suggests a systematic approach to buttock augmentation, focusing first on contour correction, then volume enhancement, and finally skin quality and cellulite improvements. Targeting specific buttock features improves outcomes by focusing on the anatomical factors that define buttock appearance.
Recommended treatment regimen	While treatment should be tailored to the patient, the following recommendations provide a consensus‐based standardized CaHA‐CMC buttock‐contouring protocol: Administer treatment over 3 sessions using 12 mL CaHA‐CMC diluted 1:1 with saline per session (total CaHA‐CMC volume = 36 mL; total saline volume = 36 mL) − Sessions should be spaced 4–6 weeks apart, with final follow‐up at 9–12 months − Use 3.0 mL CaHA‐CMC syringes (with a 10‐mL syringe for mixing) and a 22‐gauge, 70‐mm cannula for administration The panel favored treatment mapping to the outer and upper buttocks to enhance contour, using a lateral dual‐fanning injection technique.
Safety considerations	AEs are generally mild, transient, injection‐related events—consistent with other CaHA‐CMC indications. No severe AEs have been reported in published buttock augmentation studies, although the available clinical datasets remain relatively small.
	Proper training is important to ensure practitioners use CaHA‐CMC safely in the buttocks and minimize the risk of rare technique‐related complications.
Global considerations	CaHA‐CMC use in buttock augmentation varies widely with geography due to differences in practice techniques and patient preferences; future guidance must account for these global variations.

^a^ These recommendations reflect expert consensus informed by the currently available literature. They should not be interpreted as evidence‐based clinical practice guidelines because published evidence remains limited.

### 3.1 General Considerations for CaHA‐CMC‐Based Buttock Augmentation

In addition to consensus guidance (Table 3), experts highlighted the importance of defining specific anatomical features that contribute to an aesthetically pleasing buttock, including:

- **Volume**—Volume deficit identifies an area that may be a target for augmentation, typically the result of age‐related changes or genetic factors. Treating contour with CaHA‐CMC in the appropriate injection plane generally resolves mild to moderate volume deficit(s), such as hip dips, small dimples, or subtle projection improvement, thereby reducing the need for riskier, possibly surgical augmentation strategies. Typical patient requests for volume deficit correction include hip‐dips, cellulite depressions, or soft‐tissue deflation associated with significant weight loss or age‐related changes. More recently, clinicians have also reported treating patients who experience soft‐tissue volume loss and skin laxity following substantial glucagon‐like peptide 1 (GLP‐1) agonist‐associated weight loss, including buttock deflation (“Ozempic butt”). However, evidence regarding optimal management of these patients continues to evolve [12, 13]. European Union (EU) and Latin America (LATAM) experts reported using undiluted CaHA‐CMC to correct volume deficits, while others indicated nothing greater than 1:1.
- **Contour**—Buttock contouring is a cosmetic procedure that reshapes and enhances the appearance of the buttocks. This change can involve alterations in shape, volume, and projection to achieve a more youthful, rounded, or fuller look.
- **Skin Quality and Skin Laxity**—Assessment of skin quality encompasses skin tone evenness, surface evenness, firmness, and glow [4]. Skin laxity can contribute to fine lines and wrinkles in the buttocks; therefore, treating laxity may improve skin quality, and vice versa. Published studies suggest that CaHA‐CMC's biostimulatory properties may confer improvements in skin quality, and the expert panel therefore supports considering its use for treating laxity and skin quality in the buttocks with a 1:1 or, in some instances, a hyperdilute greater than 1:1 [3, 23].
- **Cellulite**—As cellulite dimples result from fibrous septa under the skin, improving overall buttock contour can lessen their appearance, making cellulite a secondary or tertiary treatment goal. Panel consensus supports the use of diluted CaHA‐CMC to treat cellulite dimpling in adult females in addition to subcision techniques [3, 18, 23, 24]. Experts noted that they use undiluted or 1:1‐diluted CaHA‐CMC to target nontethered, nonstructural cellulite. Using a cannula for CaHA‐CMC injection can disrupt fibrous septa in a manner similar to subcision, potentially further improving skin texture, as reported in the published literature [24].

### 3.2 Optimizing Outcomes and Minimizing Complications in CaHA‐CMC‐Based Buttock Augmentation

Panel recommendations indicated the importance of personalized treatment and consistent follow‐up to improve outcomes, reduce complications, and enhance patient experience in CaHA‐CMC buttock augmentation.

#### 3.2.1 Prevalence of Patient Demand and Ideal Patient Selection

Anecdotal estimates from the expert panel indicate wide regional variation in patient demand for buttock augmentation: South American‐based experts report 20 to 24 buttock‐augmentation patients per month on average, compared with 8 per month in the western USA, and 4 per month in warmer EU‐based countries, as well as the remaining US geographic regions. These estimates fluctuate with seasonal variation, when skin‐revealing warmer weather conditions are considered. Considering these estimates, ideal patients—and their relevance to achieving optimal outcomes in buttock augmentation—are summarized in Table 4. Experts emphasized that not all presenting patients are ideal candidates (e.g., those experiencing weight loss/gain of ±5 BMI points); therefore, each patient requires comprehensive assessment and expectation counseling before treatment.

**TABLE 4:** Features of an ideal patient for buttock augmentation.

Ideal patient characteristic	Relevance
Good skin quality	Healthy, firm skin supports optimal treatment results.
Adequate muscle volume	Sufficient gluteal‐muscle volume improves overall aesthetic appeal.
Athletic build or relatively good physique	A generally toned physique improves outcome.
No history of significant or rapid weight loss	A stable body weight, defined as a lifetime change in body mass index (BMI) of ≤ 5 points, helps maintain skin elasticity and reduces the risk of sagging. Patients actively undergoing significant weight loss, including those receiving GLP‐1 agonists, may benefit from delaying body contouring procedures until body weight has stabilized, as ongoing changes in soft‐tissue volume may affect aesthetic outcomes and treatment longevity [12].
No fibrotic‐contour irregularity	Subdermal fibrosis and scarring can impede uniform product placement via cannula and make it difficult to maintain the intended injection plane [24].
Younger age	Better skin elasticity and collagen production are generally observed in younger patients.
Minimal lifetime sun exposure	Reducing sun exposure lowers the risk of photoaging, skin damage, and pigmentation issues, leading to healthier skin.

#### 3.2.2 Patient Assessment

Experts noted that thorough pre‐procedure assessment helps customize treatment to patient needs and expectations, while employing multiple post‐treatment assessment methods enhances outcome evaluation.

##### 3.2.2.1 Physician‐Assessment Methods

- Patient history—A detailed evaluation of patient history, including weight trends, current or prior GLP‐1 agonist therapy, hyperpigmentation tendency, keloids, surgical and implant history, and prior aesthetic treatments, is crucial for understanding needs and expectations.
- Before‐and‐after photographs—Standardized photographs and/or special lighting considerations allow for an objective visual presentation of results; photographs are also important in expectation counseling.
- Body measurements—Tracking parameters such as body weight and buttock measurements provide useful data for correlating patient progress with treatment outcomes.
- Digitized tools—2‐ or 3‐dimensional (3D) imaging systems help visualize outcomes of buttock‐augmentation procedures (e.g., 3D tools such as Canfield's VECTRA XT or Quantificare 3D Track).
- Buttock classification—Established classification systems aid in evaluating buttock anatomy, guiding contouring decisions, and evaluating surgical outcomes [25].

##### 3.2.2.2 Patient‐Assessment Tools

- Patient‐reported outcome questionnaires—These tools allow patients to report their own post‐procedure insights, including satisfaction, quality of life, and functional changes. One example is the BODY‐Q instrument, which assesses outcomes in weight loss and body contouring [26].

Integrating physician and patient assessments provides a comprehensive foundation for buttock‐augmentation planning by aligning objective measures with patient‐reported outcomes.

#### 3.2.3 Management of Patient Expectations

Assessing patient satisfaction in aesthetic procedures is critical for improving quality of care and overall treatment outcomes [27]. High satisfaction levels are associated with greater patient motivation and engagement, including better adherence to post‐treatment instructions, consistent attendance at follow‐up visits, and more active participation in recovery—all of which contribute to improved outcomes and fewer complications [28].

Patients seeking immediate and sustained improvements need clear guidance that CaHA‐CMC's optimal effects develop gradually with dilute and hyperdilutions; pre‐ and post‐treatment photography can help patients recognize progress, thereby increasing satisfaction.

As summarized in Table 5, experts noted that most patients seeking buttock improvement lack an understanding of which specific anatomical feature (e.g., contour, volume, laxity) should be treated to derive the greatest aesthetic benefit. Following assessment, it is the practitioner's responsibility to educate and guide the patient toward the most appropriate treatment to facilitate desired outcomes. Experts stated that focusing on contouring, rather than only volumization, is important in buttock augmentation for several reasons, including:

- Enhanced aesthetics—Contouring can result in a more natural and appealing body shape, aligning with the aesthetic ideal of many patients.
- Patient satisfaction—By emphasizing contouring, clinicians can address patient expectations and concerns, reassuring them that results will be more natural and aesthetically pleasing.
- Broader appeal—A contoured buttock appeals to a wide range of individuals seeking subtle enhancements, aligning with current trends that emphasize natural beauty and balance.

**TABLE 5:** Practitioner guidance for buttock augmentation.

Example patient request	Suggested practitioner guidance	Rationale for contour focus
“I want to look rounder and more toned.”	Assess body proportions to guide personalized contouring recommendations.	Contouring refines natural aesthetics and improves overall silhouette.
“I just want to look bigger overall.”	Emphasize balanced aesthetics to achieve desired contour.	Improvement in contour and silhouette is important, rather than volume only.
“How can I achieve that ‘lifted look’ without going overboard?”	Describe CaHA‐CMC as a minimally invasive option for contour and volume enhancement.	A well‐contoured buttock enhances perceived lift and youthfulness.
“I want a healthy, natural appearance.”	Emphasize subtle contouring to maintain a natural appearance.	Balanced contouring supports a natural aesthetic result.
“How do recovery times for minimally invasive treatments compare with those of surgical procedures?”	Highlight how CaHA‐CMC allows patients to resume normal activities sooner due to minimal tissue trauma compared with surgical incisions.	Patients favor procedures with efficient recovery and natural‐looking results, as achieved with CaHA‐CMC‐based buttock contouring.

Thus, prioritizing buttock contour may yield secondary improvements in other aesthetic concerns, including cellulite, skin laxity, or overall skin quality/appearance.

#### 3.2.4 Patient‐Centered Considerations for Optimal Treatment Outcomes

Effective buttock augmentation requires a patient‐centered approach to tailor evaluation and treatment to individual needs. The following principles help ensure safe, personalized, and outcome‐focused care:

- Individualized treatment—Develop plans that reflect each patient's anatomy, lifestyle, budget, and goals.
- Clear communication—Maintain open dialog to understand patient preferences, address concerns or anxiety, and align expectations.
- Population‐specific needs—Adapt treatment strategies to unique considerations among different patient groups (e.g., males may experience different outcomes with buttock treatments due to variations in body composition and fat distribution).
- Ongoing monitoring—Use regular follow‐up visits to evaluate outcomes, gather patient feedback, and adjust treatment plans as needed.

### 3.3 Recommended Treatment Regimen for CaHA‐CMC‐Based Buttock Augmentation

CaHA‐CMC's ability to provide immediate volume replacement, followed by progressive neocollagenesis, makes it an ideal candidate for improving buttock contour, shape, and skin quality [3, 4].

#### 3.3.1 Injection Preparation

Several CaHA‐CMC formulations are used for different aesthetic purposes, including:

− Undiluted CaHA‐CMC
− Standard dilution: 1:1 CaHA‐CMC
− Hyperdiluted: at least 1:2 CaHA‐CMC

Aqueous dilution reduces CaHA‐CMC's elastic modulus and increases its liquidity and spread. Laboratory rheological and perfusion‐model studies suggest that increasing dilution may reduce proximal occlusive potential by altering the product's rheological properties [5, 29, 30]. Although experimental models support these findings, their clinical relevance remains to be established.

Based on the available evidence and panel experience in buttock contouring, a 1:1 CaHA‐CMC‐saline dilution was considered the preferred starting approach for most patients, as it may improve tissue spread without demonstrable material distribution beyond the intended treatment plane [31], enhance initial volumization, and ensure optimal CaHA‐particle distribution for fibroblast and nucleogenesis stimulation [5, 15, 29, 32]. This ratio provides immediate, visible improvement with progressive biostimulatory volumization [5], and can also address secondary aesthetic concerns, such as cellulite appearance or skin quality.

When using diluted CaHA‐CMC, the panel suggested that practitioners should consider the total injection volume (product + diluent) per patient and per session. Due to variation in off‐label and approved uses, syringe sizes vary with geography—1.5‐mL syringes are common in the US, whereas 1.5‐ or 3‐mL syringes are frequently used in the EU and LATAM. Thus, per‐patient volumes should be reported as total volume of CaHA‐CMC administered (in milliliters or cubic centimeters) with additional considerations for volume per treatment to ensure global transparency. Importantly, product mixtures must be fully homogeneous to prevent extrusion clumping, backflow, and alterations in flow behavior [24].

#### 3.3.2 Injection Technique

Patient positioning in buttock augmentation may influence aesthetic outcomes. Practitioners may assess, mark, and treat patients standing or supine; however, the panel preferred standing pre‐treatment marking because it more accurately represents gravity‐dependent contour issues and displays projection (i.e., from the patient's perspective).

**FIGURE 1:**
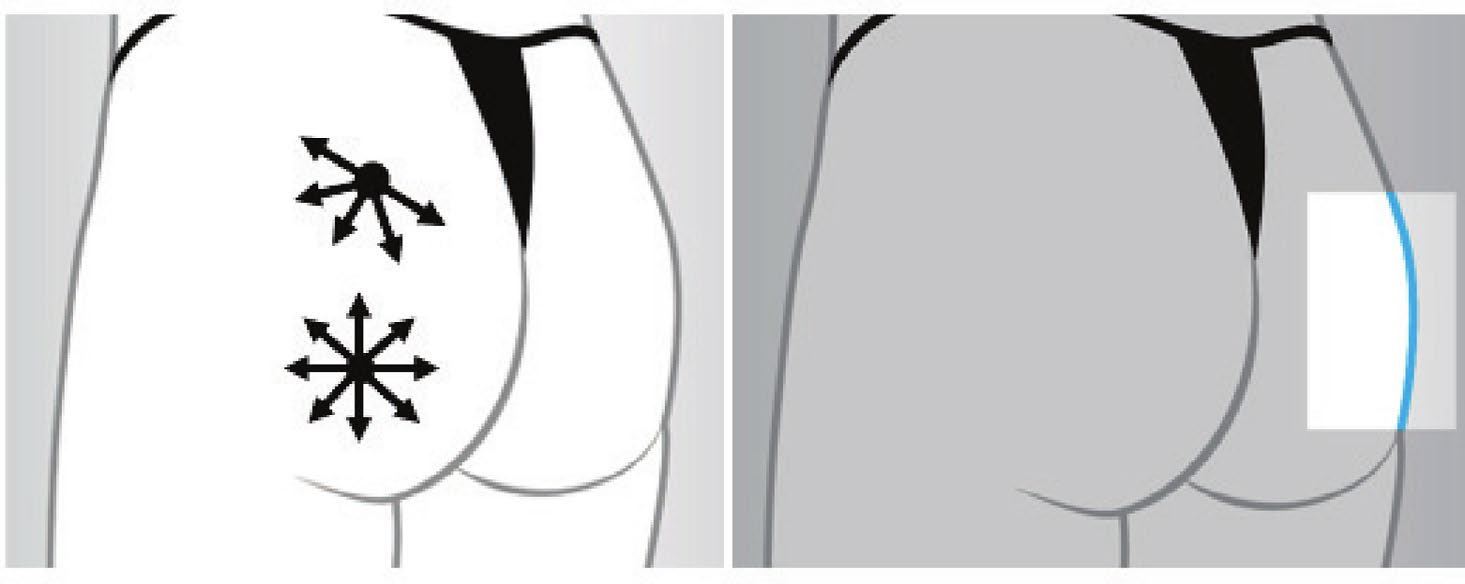
Proposed buttock contour treatment map and area of assessed improvement (based on expert consensus discussion).

Preferred CaHA‐CMC‐injection sites for buttock contouring are the upper outer quadrants, as recommended by experts in a new targeted contour map (Figure 1). Using a cannula enables precise placement, broad and balanced product distribution, reduced injection‐site trauma, and greater efficiency [3, 24]. Injection techniques, including linear threading, asterisk fanning, and cross‐hatching, help ensure even CaHA‐CMC distribution.

During injection, CaHA‐CMC may migrate retrograde and pool at the needle entry/port site. Gentle massage or compression of the port site may help prevent this accumulation, thereby reducing the risk of nodules. Ensuring thorough dispersion of the product throughout the treatment area is considered important for achieving uniform distribution and minimizing lumps or nodules.

Identifying key anatomical landmarks—including the buttock quadrants, gluteal sulcus, and gluteal midline—is critical during treatment. When performing deeper injections without a cannula, caution is required to avoid injury to critical vasculature (i.e., the inferior gluteal vein) and neural structures.

Topical lidocaine may be applied at the entry point to reduce procedural discomfort. Following treatment, gentle massage and cold packs help improve comfort.

#### 3.3.3 Injection Volume

Although injection volumes vary across regions—with US practitioners typically reporting higher volumes than those in Argentina, Spain, or Italy—selecting the appropriate volume for each patient remains essential for optimal buttock‐contouring results. The expert panel proposed a standardized injection volume and treatment plan (Table 6), paired with a targeted contour map (Figure 1), to achieve the best outcomes.

**TABLE 6:** Recommended injection volumes for CaHA‐CMC in buttock contouring.

	Session 1 (mL per side^a^)	Session 2 (mL per side^a^)	Session 3 (mL per side^a^)	Total (mL per side^a^)
CaHA‐CMC	12 mL (6/6)	12 mL (6/6)	12 mL (6/6)	36 mL (18/18)
Saline	12 mL (6/6)	12 mL (6/6)	12 mL (6/6)	36 mL (18/18)
CaHA‐CMC + saline	24 mL (12/12)	24 mL (12/12)	24 mL (12/12)	72 mL (36/36)

^a^ (#/#) = diluted Radiesse volume to each buttock cheek.

#### 3.3.4 Post‐Treatment Care

Experts noted that patients may experience mild, transient bruising, edema, or tenderness following treatment. To support optimal results and minimize discomfort, patients should use gentle massage at home for 1 week. Patients should avoid applying pressure to treated areas and refrain from strenuous activities that heavily engage the buttock muscles for the first few days.

#### 3.3.5 Treatment Schedule

CaHA‐CMC‐based buttock augmentation generally requires multiple (2–4) treatment sessions, spaced 4 to 6 weeks apart, with follow‐up at 9 to 12 months. The longer follow‐up interval allows adequate time to evaluate CaHA‐CMC's biostimulatory volumizing effects, as the improvement is progressive [33, 34].

#### 3.3.6 Safety Considerations

The short‐ and long‐term safety of CaHA‐CMC is well documented for its approved indications. Reported adverse events (AEs) are typically minor and comprise transient administration‐site conditions, including edema, erythema, pain, hemosiderin staining, bruising, and hyperpigmentation [2, 6, 8, 9]. Although vascular occlusion is uncommon, it is a potentially serious complication of injectable filler procedures. The recommended subdermal injection plane lies superficial to the major gluteal vessels and may help reduce the risk of vascular injury. However, the gluteal region contains important vascular structures that require careful attention to anatomy. Experimental studies suggest that increasing product dilution may reduce proximal occlusive potential [5, 30]. Observational clinical studies have shown lower rates of vascular occlusion with microcannulas than with needles in aesthetic filler procedures [35]. Nevertheless, vascular complications cannot be completely avoided. Careful patient selection, detailed knowledge of gluteal anatomy, proper injection technique, and appropriate practitioner training remain essential for minimizing risk.

As reported in the clinical literature (Table 1), AEs associated with CaHA‐CMC‐based buttock treatment are similar to those observed with other indications. According to Teodoro et al. (2023) [21], more than 100 CaHA‐CMC buttock‐augmentation procedures were performed over 5 years without severe adverse events and when using relatively large CaHA‐CMC volumes in a single session (i.e., up to 30 mL CaHA‐CMC and 87 mL saline at 1:1, 1:2, and 1:4).

In summary, current evidence supports a favorable safety profile for CaHA‐CMC in buttock augmentation; however, practitioners should remain vigilant during treatment to minimize the risk of complications.

### 3.4 Limitations

Many recommendations in this consensus document are based on a small number of prospective and retrospective studies, case reports and case series, and expert clinical experience because high‐quality comparative evidence for CaHA‐CMC buttock augmentation remains limited. As additional prospective studies become available, these recommendations should be refined and updated accordingly.

### 3.5 Future Directions

As the use of CaHA‐CMC for buttock augmentation continues to expand, comprehensive studies evaluating its safety and effectiveness will be essential. Future initiatives to optimize injection protocols and techniques, improve patient outcomes, and minimize complications may include advanced practitioner training, integration of new technologies for patient assessment and product administration, and greater customization of individual treatment plans. Prospective comparative studies and randomized clinical trials are needed to strengthen the evidence base supporting many of the recommendations presented in this consensus statement.

## 4 Conclusion

This global expert consensus provides practical guidance on the use of diluted CaHA‐CMC for buttock augmentation and contouring based on the currently available published evidence and expert clinical experience. These consensus‐based recommendations are intended to support safe and effective treatment with CaHA‐CMC treatment by appropriately trained practitioners. Achieving reproducible outcomes relies on adherence to these principles together with a patient‐centered approach that emphasizes careful patient selection and assessment, expectation management, and anatomically guided, individualized treatment.

When used appropriately, the available evidence suggests that CaHA‐CMC has a favorable safety profile. However, published data supporting its use in buttock augmentation remain limited. As clinical experience in buttock augmentation and contouring continues to expand, additional prospective comparative studies and systematic collection of real‐world outcome data will be needed to further optimize outcomes, enhance patient safety, refine treatment protocols, and strengthen the evidence base to support consistent, high‐quality practice worldwide.

## Author Contributions

All authors participated in a virtual meeting, in which discussions were informed by a review of supporting peer‐reviewed publications, clinical practice, and clinical studies. G.C., K.K.D., and Z.P.L. contributed foundational research that informed the discussions. All authors contributed to the development of the consensus statements and recommendations, and participated in the writing, reviewing, and editing of the manuscript for important intellectual content. All authors have read and approved the final manuscript.

## Funding

Medical writing support was funded by Merz Aesthetics.

## Ethics Statement

The authors confirm that the ethical policies of the journal, as noted on the journal's author guidelines page, have been adhered to. No ethical approval was required as this is a review article with no original research data.

## Conflicts of Interest

Merz Aesthetics had no role in the development of consensus statements. All content, interpretations, and final decisions reflect the consensus of the author group and their contributions.

## Data Availability

Data sharing is not applicable to this article, as no data sets were generated or analyzed during the current study.
